# A Correlation of Blood Panel Results and Histologically Confirmed Appendicitis

**DOI:** 10.7759/cureus.10641

**Published:** 2020-09-25

**Authors:** David Keohane, Peter O'Leary, Matthew Nagle, Kim Cichelli, Tom McCormack

**Affiliations:** 1 General Surgery, University Hospital Kerry, Tralee, IRL; 2 General Surgery, Cork University Hospital, Cork, IRL; 3 Orthopaedics, Cork University Hospital, Cork, IRL; 4 Internal Medicine, Medical University of South Carolina, Charleston, USA

**Keywords:** appendicitis, histology, blood panel, wbc, neutrophils, crp

## Abstract

Background

Appendicitis is the most common indication for emergency surgery in the world. There is no one laboratory or radiological test that is used to diagnose it. Various routine and novel blood markers have been identified, however none have proved to be conclusive. The aim of this study was to combine routine blood markers to increase the sensitivity and specificity in diagnosing histologically confirmed appendicitis.

Methods

We retrospectively reviewed the theatre logs for the calendar year of 2015 to identify all of the appendectomies which were performed. We reviewed all of the admission bloods for the patients - including their white blood cell (WBC) count, their neutrophil count, and their C-Reactive protein (CRP) value. We also reviewed all of the histology to identify the inflamed appendices, and analysed all of this information together.

Results

The neutrophil count is the most sensitive of the three blood markers with a score of 82%. It has a specificity of 63%. The CRP value is the most specific of the three blood markers with a value of 67% and a sensitivity of 76%. WBC has a sensitivity of 75% and a specificity of 63%. Combining all of the blood values (i.e. elevated white blood cell count or elevated neutrophil count or elevated CRP) demonstrates a sensitivity of 96% and a specificity of 45%.

Conclusion

Combining routine admission blood markers (WBC, neutrophil count, and CRP) can assist in diagnosing appendicitis in unwell patients with abdominal pain.

## Introduction

Appendicitis is the most common indication for emergency surgery [1,2]. The appendix is one of the most studied organs in surgery, however we are no closer to developing a single test (either laboratory or radiologically) that will definitely diagnose acute appendicitis. Standard blood tests such as white blood cell (WBC) count, neutrophil count, C-reactive protein (CRP), bilirubin, alanine transaminase (ALT), and albumin have been used as markers for acute appendicitis, and in recent years novel blood markers have been proposed such as procalcitonin, interleukin-6 (IL-6), serum amyloid-A (SAA), granulocyte colony-stimulating factor (G-CSF) and calprotectin as markers for acute appendicitis [3-10].

Ratios of blood results have also been studied including white cell lymphocyte ratio, white cell neutrophil ratio, and neutrophil ratio [11] to differentiate between uncomplicated and complicated appendicitis. Significant issues with sensitivity and specificity exist for all of these individual tests. A systematic review by Kabir et al. identified multiple studies that have combined various blood markers (including WBC count, neutrophil count, and CRP) in order to try and increase the sensitivity and specificity of these commonly available blood tests. While the results of these combined blood panels have been encouraging, Kabir does note that more research is needed in this area [10].

Therefore, acute appendicitis largely remains a clinical diagnosis. This can have many consequences. A late or missed diagnosis can result in complications such as perforation, abscess formation or, in extreme cases, death [1]. It can also be over-diagnosed with hospitals reporting negative appendectomy rates of 3.3% to 26.7% [12-18]. Every negative appendectomy is an unnecessary surgical procedure for that patient. It exposes the patient to risks associated with surgery - such as anaesthetic, bleeding, peri-operative infection, abdominal viscera perforation, pain, scarring, and the development of a post-operative collection and adhesions, as well as the risks associated with hospital admission such as hospital-acquired infection, deep vein thrombosis, and pulmonary embolism. It also has the secondary effect of using already limited hospital resources, such as inpatient beds and theatre time.

Thus this study sought to analyse the usefulness of widely available inflammatory biomarkers in diagnosing acute appendicitis. Inflammatory biomarkers were also analysed in different combinations to establish if this would increase the sensitivity and specificity of diagnosing histologically inflamed acute appendicitis.

## Materials and methods

We retrospectively reviewed the data from all patients who had appendectomies carried out in a regional Irish hospital in 2015. The hospital serves a large geographic area with a county population of approximately 150,000 people (49.6% male and 50.4% female). Theatre log books were reviewed to identify every appendectomy that took place in the hospital. Data captured included patient demographics, serological results, radiological investigation results, type of operative technique, and histological findings.

The blood results (WBC, neutrophils, and CRP) and histology for all of these patients were reviewed and analysed using an Excel spreadsheet. A review of the ultrasound (US) imaging done on each of these patients was also carried out. Normal CRP levels are considered as <=5 mg/L - regardless of age of patient. CRP levels of >5 mg/L are considered high. Reference ranges for normal WBC serum levels are 4 - 11 (x10^9^/L) in the adult population. Reference ranges for normal WBC serum levels are 4 - 14.5 (x10^9^/L) in the paediatric population (age <=12 years old). WBC <4 (x10^9^/L) is considered low for the adult and paediatric population. WBC >11 (x10^9^/L) is considered high for the adult population. WBC >14.5 (x10^9^/L) is considered high for the paediatric population (age >12 years old). The normal reference range for the absolute neutrophil count (ANC) is 1.5 - 8 (x10^3^/mL) for the adult and paediatric population. ANC is considered low if it is <1.5 (x10^3^/mL). ANC is considered high if it is >8 (x10^3^/mL).

Descriptive statistics were used to assess patient demographics and blood results against the histological findings. The Pearson Chi2 test was used to analyse the variables. A p-value of less than 0.05 was taken to be statistically significant. All statistical analyses were performed using the software Stata/IC 13.1 (StataCorp., College Station, TX, USA) for Mac (64-bit Intel).

## Results

Two hundred forty-one appendectomies were performed between January 1, 2015 and December 31, 2015. One hundred seventy-five (73%) appendixes had histopathological findings of inflammation, 173 of these (98.8%) were reported as inflamed. One appendix was found to have a mucinous neoplasm, and one appendix contained a neuroendocrine carcinoid tumour. Sixty-six appendixes had normal histopathological findings - this includes one appendix that had a faecolith noted on analysis. The negative appendectomy rate was 27.4%.

A breakdown of the demographics and operative method can be found in Table 1. One hundred thirty-one (54%) of the patients were male and 110 (46%) were female. In total, 54 (22%) patients who underwent an appendectomy were aged 12 or under, 62 (26%) patients were aged between 13 and 18, and 125 (52%) patients were over 18 years old. The mean age of all 241 patients was 25.4 years.

**Table 1:** Breakdown of inflamed versus non-inflamed appendixes

Category	Sub-category	Total n	Inflamed n (%)	Non-inflamed n (%)	p-value
Gender	Male	131	96 (73%)	35 (27%)	0.8
	Female	110	79 (72%)	31 (28%)	
Age	<= 12	54	38 (70%)	16 (30%)	0.52
	13-18	62	41 (66%)	21 (34%)	0.39
	> 18	125	96 (77%)	29 (23%)	0.33
Operative Method	Laparoscopic	177	122 (69%)	55 (31%)	0.03
	Open	64	53 (83%)	11 (17%)	

One hundred seventy-seven (73%) appendixes were removed laparoscopically, and 64 (27%) were removed as an open procedure. Of the 64 open appendectomies performed, 42 (66%) were performed on the paediatric population (<=18 years old) even though they represented 48% of the total number of appendectomies.

CRP analysis

A CRP was done on 173 of the 175 patients who had a histologically inflamed appendix, and 61 of the 66 normal appendixes. The CRP was normal in 41 (24%) inflamed appendixes, and elevated in 132 (76%) inflamed appendixes. Out of the 66 non-inflamed appendixes, CRP was normal in 41 (67%) patients, and was elevated in 20 (33%) patients. The mean CRP values for the inflamed appendixes was 59.2, and it was 22.5 in the normal appendixes. The results are summarised in Table 2 below.

**Table 2:** Correlation of histology and C-Reactive protein (CRP) results * 2 patients did not have a CRP taken ^ 5 patients did not have a CRP taken

	Histologically Inflamed*	Histologically Non-inflamed^	Total	p-value
Elevated CRP	132	20	152	0.0001
Normal CRP	41	41	82	
Total	173	61	234	

**Figure 1:**
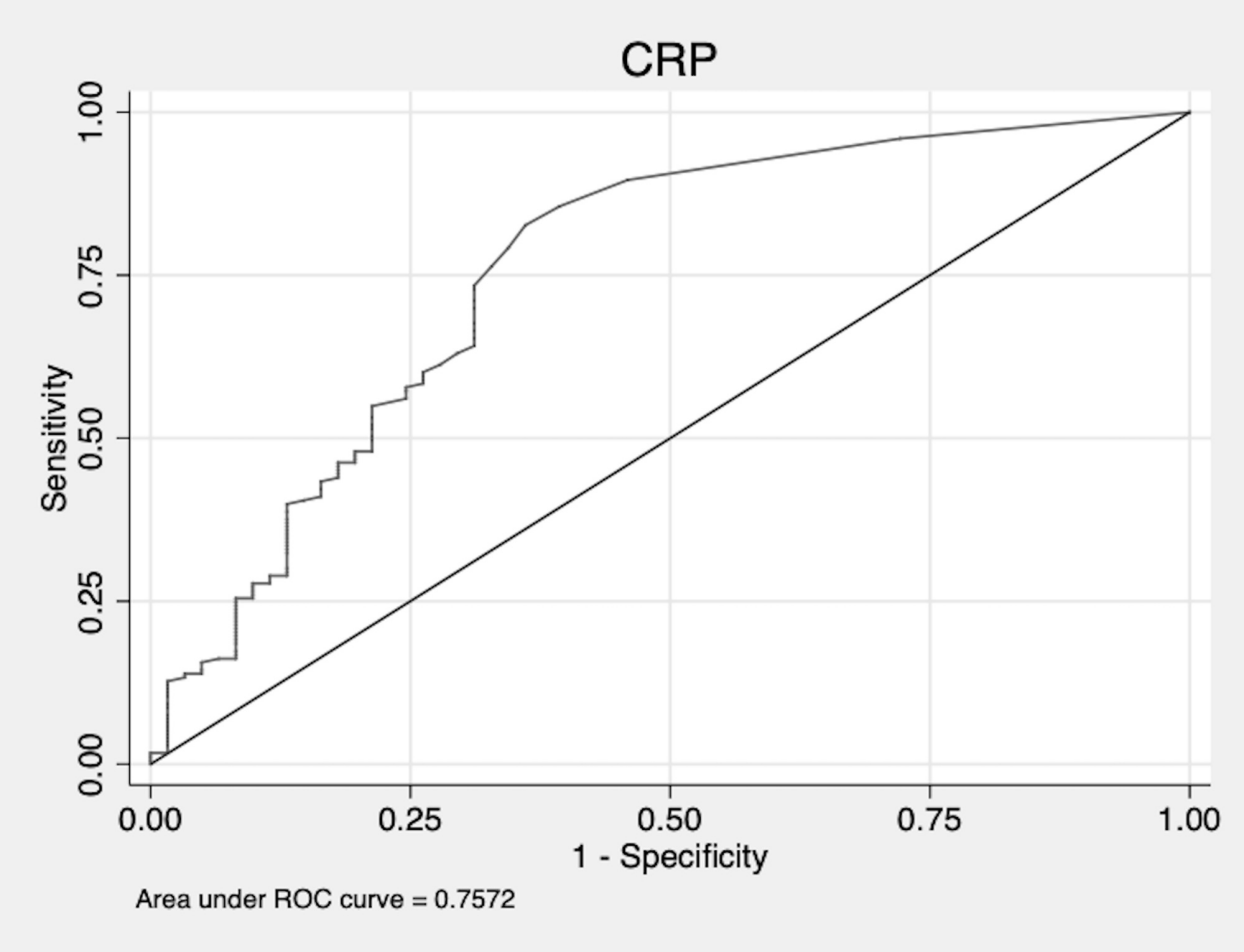
CRP AUC CRP: C-Reactive protein, AUC: area under the curve

The sensitivity of using an elevated CRP to diagnose a histologically inflamed appendix was 76%. The specificity was 67%. The receiver operating characteristic (ROC) area was 0.72. The positive predictive value (PPV) of an elevated CRP was 87%. The negative predictive value (NPV) was 50%. The area under the curve (AUC) is displayed in Figure 1.

WBC analysis

A full blood count (FBC) was done on 174 of the 175 patients who had a histologically inflamed appendix, and 64 of the 66 normal appendixes. The WBC was normal for 43 (25%) inflamed appendixes, low for two (1%) of them and elevated in 129 (74%) inflamed appendixes. Out of the 64 non-inflamed appendixes, WBC was normal in 40 (63%) patients and elevated in 24 (37%) patients. The mean WBC count for the inflamed appendixes was 15.1 and 10.2 for the normal appendixes. The results are summarised in Table 3 below.

**Table 3:** Correlation of histology and white blood cell (WBC) results * 1 patients did not have a WBC taken ^ 2 patients did not have a WBC taken

	Histologically Inflamed*	Histologically Non-inflamed^	Total	p-value
High/Low WBC	131	24	155	0.0001
Normal WBC	43	40	83	
Total	174	64	238	

**Figure 2:**
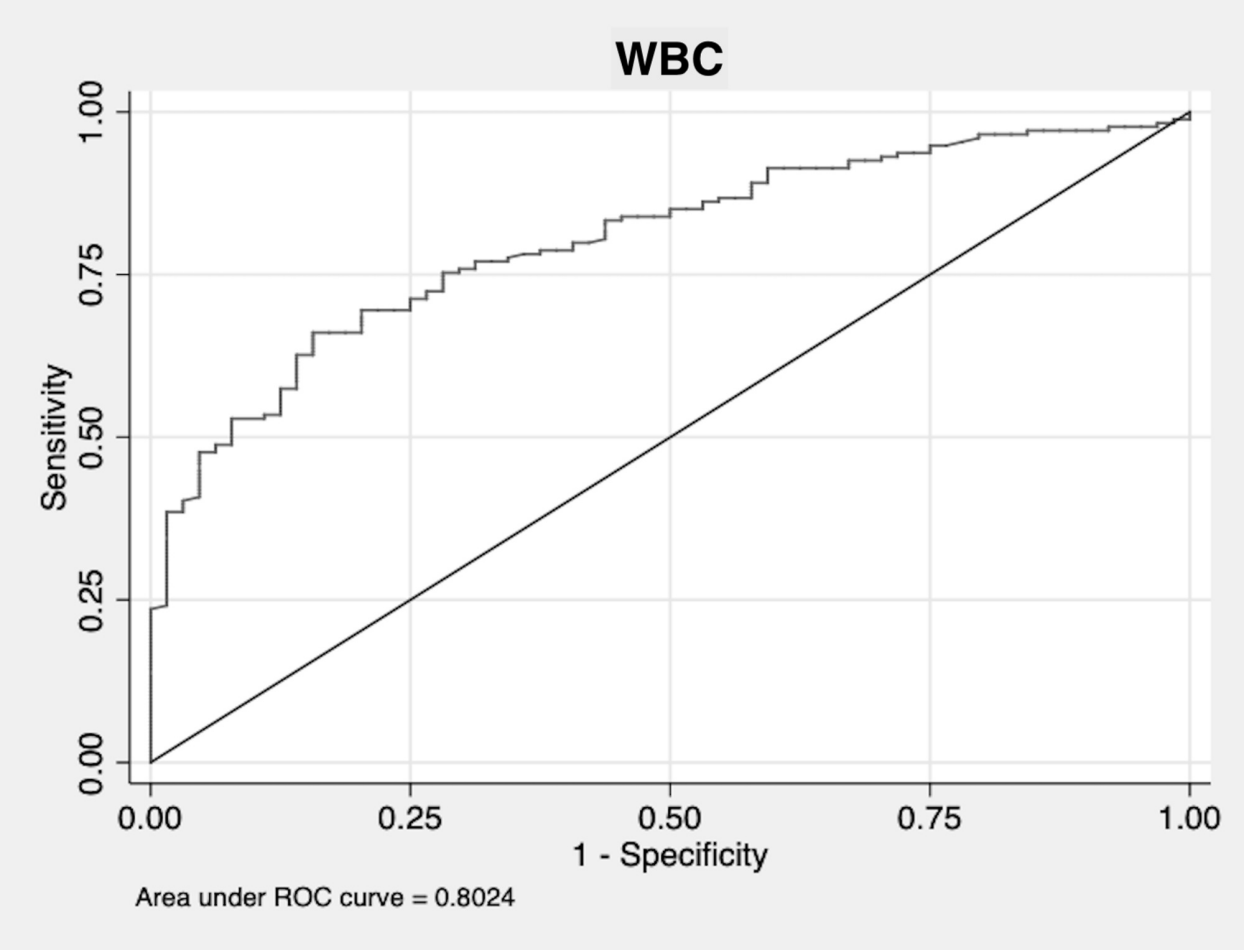
WBC AUC WBC: white blood cell, AUC: area under the curve

The sensitivity of using a high or low WBC to diagnose a histologically inflamed appendix was 75%. The specificity was 63%. The ROC area was 0.69. The positive predictive value of a high or low WBC was 85%. The negative predictive value was 48%. The AUC is displayed in Figure 2.

Neutrophil analysis

The neutrophil count was normal for 31 (18%) inflamed appendixes, and elevated in 143 (82%) inflamed appendixes. Out of the 64 non-inflamed appendixes where an FBC was done, neutrophil count was normal in 40 (63%) patients and elevated in 24 (37%) patients. The mean neutrophil count for the inflamed appendixes was 12.4 and 7.1 for the normal appendixes. The results are summarised in Table 4 below.

**Table 4:** Correlation of histology and neutrophil count * 1 patients did not have a neutrophil count taken ^ 2 patients did not have a neutrophil count taken

	Histologically Inflamed*	Histologically Non-inflamed^	Total	p-value
High Neutrophils	143	24	167	0.0001
Normal Neutrophils	31	40	71	
Total	174	64	238	

**Figure 3:**
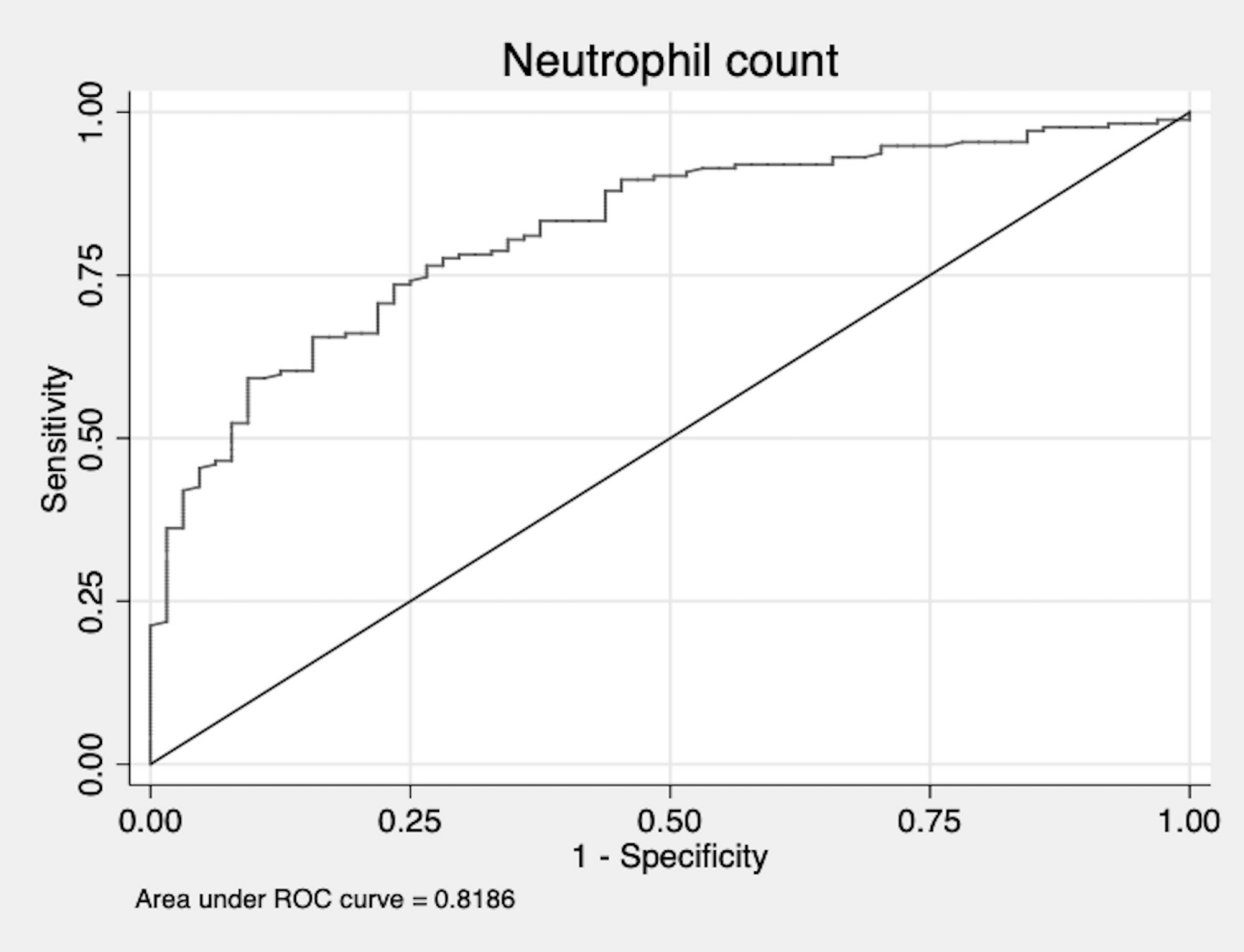
Neutrophil AUC AUC: area under the curve

The sensitivity of using a high neutrophil count to diagnose a histologically inflamed appendix was 82%. The specificity was 63%. The ROC area was 0.72. The positive predictive value of a high neutrophil count was 86%. The negative predictive value was 56%. The AUC is displayed in Figure 3.

Histology and combined blood panel results

Out of the 175 histologically inflamed appendixes, 174 had either an FBC or CRP taken. Ninety-nine (57%) patients had a combination of elevated CRP, leukocytosis (or leukopenia), and neutrophilia. Seven patients with histologically confirmed appendicitis had a normal CRP, WBC count and neutrophil count. Five of these seven patients were under the age of 18. The sensitivity of either a high CRP, leukocytosis or neutrophilia was 96%. The specificity was 45%. The ROC area was 0.71. The positive predictive value of abnormal blood results was 83%. The negative predictive value was 81%. These results are summarised in Table 5. 

**Table 5:** Correlation of histology and any raised blood marker * 1 patient did not have either an FBC or a CRP taken ^ 2 patients did not have either an FBC or CRP taken FBC: full blood count, WBC: white blood cell, CRP: C-reactive protein

	Inflamed*	Non-inflamed^	Total	p-value
Elevated CRP OR High WBC Count OR High Neutrophils	167	35	202	0.0001
Normal Bloods	7	29	36	
Total	174	64	238	

A summary of all of the blood results are found in Table 6.

**Table 6:** Summary of findings for each blood results WBC: white blood cell, CRP: C-reactive protein, PPV: positive predictive value, NPV: negative predictive value

	Sensitivity	Specificity	PPV	NPV	ROC AUC
Elevated CRP	76.3% (95% CI: 69.3-82.4%)	67.2% (95% CI: 54.0-78.7%)	86.8% (95% CI: 80.4-91.8%)	50.0% (95% CI: 38.7-61.3%)	0.72 (95% CI: 0.65-0.78)
Elevated WBC	75.3% (95% CI: 68.2-81.5%)	62.5% (95% CI: 49.5-74.3%)	84.5% (95% CI: 77.8-89.8%)	48.2% (95% CI: 37.1-59.4%)	0.69 (95% CI: 0.62-0.76)
Elevated Neutrophils	82.2% (95% CI: 75.7-87.6%)	62.5% (95% CI: 49.5-74.3%)	85.6% (95% CI: 79.4-90.6%)	56.3% (95% CI: 44.0-68.1%)	0.72 (95% CI: 0.66-0.79)
Elevated CRP OR WBC count OR Neutrophil count	96.0% (95% CI: 91.9-98.4%)	45.3% (95% CI: 32.8-58.3%)	82.7% (95% CI: 76.7-87.6%)	80.6% (95% CI: 64.0-91.8%)	0.71 (95% CI: 0.64-0.77)

## Discussion

A routine blood panel is taken on all patients that present to the emergency department (ED) with any infective or inflammatory symptoms. This includes an FBC, CRP and a metabolic/renal profile. At present, a single blood or urine marker for acute appendicitis does not exist, and the existing blood markers used are not sensitive or specific enough for it [4]. Our research has demonstrated that combining commonly used blood markers can increase their sensitivity to 96% in the setting of acute appendicitis.

A meta-analysis by Kabir et al. has demonstrated that a WBC count in isolation is not a good marker to use for diagnosing appendicitis as it can be elevated in response to any inflammatory condition [10]. Shogilev et al. describe multiple different cut-off values ranging from 9.4 to 14.6 (x109/L) with no clear recommendation on which one is best in the context of identifying acute appendicitis [3]. Of note, the commonly accepted range for a normal WBC count is 4 to 11 (x109 /L) so some of the studies included in Shogilev’s analysis were using WBC counts that would be classified as normal. By accepting low threshold WBC counts, we would expect the sensitivity to increase but the specificity to decrease - making the test less useful. We found that WBC count had the lowest sensitivity (75%) and specificity (63%) of the three blood results we examined. They were within the given range for sensitivity (65-85%) and specificity (32-82%) in the systematic review done by Shogilev et al. [3].

Neutrophil count can also be used to help identify an inflamed appendix [4,8] although it is not given as much consideration in the literature compared to WBC count. It can also be elevated in any infective condition and usually indicates a bacterial etiology. Kabir et al. reviewed 10 publications that examined the role of polymorphonuclear (PMN) cells and found a sensitivity and specificity of between 71-89% and 48-80% in the diagnosis of acute appendicitis [10]. This range of values is attributable to the fact that different papers used different cut-off values for measuring sensitivity and specificity. Those values ranged from 7 to 13. In our research, we used a cut-off value of over 8 to indicate an elevated neutrophil count. Using that, we found that the neutrophil count had the highest sensitivity (82%) of all of the blood results we looked at and it had the joint highest specificity (64%) - along with CRP.

CRP also has a variable role in the literature when it comes to appendicitis. It has been noted in the systematic review by Kabir et al. that CRP is better for detecting complicated or late-stage appendicitis as it is a lag indicator and is less useful for early-stage appendicitis [10]. Other papers have also demonstrated that it is less useful for acute appendicitis but significant elevation is suggestive of abscess or perforation [4,8]. Zani et al. found that a CRP cut-off value of 40 identified one-third of paediatric patients with simple appendicitis but two-thirds of complicated appendicitis. However when they increased their CRP cut-off value to 80, they only identified 40% of their perforated paediatric appendixes [7]. These values differ significantly from Van den Worm et al. who found that a CRP cut-off value of 215 was fair in helping to diagnose complex appendicitis [9] - although it should be noted that the casemix of their study was different to Zani. The mean CRP value for histologically inflamed versus normal appendixes in our study was 59.7 versus 23.7. These values were similar to mean CRP values reported by Al-Abed et al. of 73 versus 32 for inflamed and normal appendixes [5]. The CRP figures for the sensitivity and specificity in our research were 77% and 64% - again these values fell within the range quoted by Shogilev et al. of 65-85% and 59-73% [3].

Some research has been done in the field of combining serological markers to increase sensitivity and specificity for diagnosing acute appendicitis. Birchley demonstrates that combining WBC count, neutrophil count, and CRP gives an odds ratio of 18 when trying to identify acute appendicitis - although it should be noted that this is only applicable when all of those tests are abnormal [4]. Farooqui et al. looked at WBC, bilirubin, CRP, and ALT and noted that combining these biomarkers increased the predictive value when trying to discriminate between acute appendicitis and non-appendicitis [6].

Our findings when the three different blood results were combined were promising. It is worth emphasizing that we combined any abnormal WBC count, neutrophil count, or CRP value - we did not specify that all three results had to be abnormal. They demonstrated a sensitivity for detecting a histologically confirmed appendicitis of 96% - this is encouraging as it demonstrates that if an appendix is histologically inflamed, it is highly likely to be reflected in at least one of the blood results. Therefore if WBC count, neutrophil count, and CRP are all normal, then it is likely that the appendix is normal - if clinical suspicion persists, then either imaging or a diagnostic laparoscopy should be performed. Although the specificity was only 44% - this is not surprising as these blood markers could be raised due to any inflammatory or infective condition. This is similar to findings published by Andersson who found that a combination of WBC >10 and a CRP >8 gave an accuracy of 0.96 (area under the curve) for diagnosing appendicitis [19].

The US sensitivity (59%) and specificity (85%) results in our study were not encouraging - but they are similar to a study by Yuan et al. who reported figures of 50% and 98.5% [20] and D’Souza et al. who reported figures of 51.8% and 81.4% [15] respectively. These figures differ significantly from Gracey et al. who reported sensitivity and specificity of 93.8% and 90.6% [21]. It is hard to reconcile the discrepancy between the different studies, however it may be attributable to operator experience. Our figures imply that US may be a better screening tool, which is then combined with another imaging modality, such as CT, to try and reduce the negative appendectomy rate. At best, it should be combined with the blood results, history, and clinical exam in deciding whether or not to bring a patient to theatre.

This study has several limitations. These include the fact that it was a retrospective study. No clinical information was captured about any patient. No complications for any patient were included. No information was recorded about the use of CT scans for patients in this study. We would recommend a more detailed future study which would include clinical details for the patient prompted the decision to bring the patient to theatre.

## Conclusions

Appendicitis is the most common surgical emergency in the world. There is no one diagnostic blood test or radiographical scan which has been shown to categorically prove appendicitis. The gold standard for diagnosing it is to examine the histology post-operative removal. Several standard blood tests have been examined and multiple novel blood tests have been examined to try and identify a better way of diagnosing it. The existing literature suggests combining routinely available blood tests may be a way forward. Our research builds on that premise and demonstrates that while elevated WBC, neutrophil count, and CRP in isolation can be helpful in aiding the clinical diagnosis of acute appendicitis, they are more useful when combined.
